# Ultrafast Excited-State Dynamics of a Porphyrin–OPE Conjugate: Energy Transfer and Aggregation Effects

**DOI:** 10.1021/acs.jpcc.5c07515

**Published:** 2025-11-26

**Authors:** Francesco Tumbarello, Federico Toffoletti, Chiara Maria Antonietta Gangemi, Mariagrazia Fortino, Adriana Pietropaolo, Anna Barattucci, Luigi Monsù Scolaro, Maria Angela Castriciano, Elisabetta Collini

**Affiliations:** † Department of Chemical Sciences, University of Padova, via Marzolo 1, Padova 35131, Italy; ‡ Dipartimento di Scienze Chimiche, Biologiche, Farmaceutiche ed Ambientali (ChiBioFarAm), 18980 Università degli Studi di Messina, Messina 98166, Italy; § Dipartimento di Scienze della Salute, Università di Catanzaro, Viale Europa, Catanzaro 88100, Italy

## Abstract

Understanding the ultrafast dynamics of porphyrin-based conjugates is crucial for their applications in areas such as photovoltaics, molecular electronics, and biocompatible technologies. In this study, we investigate the excited-state dynamics of a porphyrin–oligo­(phenylene ethynylene) (OPE) conjugate with β-d-glucoside termination using femtosecond transient absorption spectroscopy in both monomeric and aggregated states. Our findings reveal that in the monomeric form, rapid energy transfer (200 ps) from the OPE unit to the porphyrin core occurs. When aggregation is induced, we observe a significant shortening of the relaxation dynamics, suggesting that the new supramolecular interactions at play strongly influence the overall photophysical behavior of the system, potentially allowing for effective control of the energy transfer processes.

## Graphical abstract

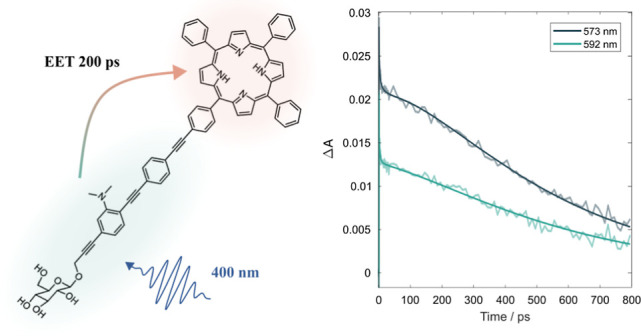

## 1 Introduction

Porphyrin-based supramolecular systems have garnered significant attention due to their versatile optical and electronic properties, making them essential candidates for applications in photodynamic therapy, imaging, photovoltaics, and photonics.^1–3^ Their ability to self-assemble into well-defined nanoaggregates via noncovalent interactions, such as π-stacking and hydrogen bonding, plays a crucial role in their functionality, making them highly valuable materials.^4^

In previous research, a new amphiphilic system was designed; it featured a free-base porphyrin macrocycle (tetraphenyl porphyrin, TPP), monosubstituted at the meso-position with an oligophenylenethylene (OPE)^5^ residue bearing a dimethylamino substituent on one of the aromatic residues and a β-d-glucoside termination.^6^ The single functionalization of the macrocycle with the rigid OPE spacer aimed to build a novel nonhindered amphiphilic porphyrin and investigate the influence of monosubstitution on its aggregation properties.^6^ The introduction of the chromophore OPE also enables the investigation of the mutual influence and electronic communication with the porphyrin, which could be further enhanced by the presence of the electron-donating dimethyl amino group. The dimethylamino substituent on the OPE plays a dual role in the molecular design: it acts as an electron-donating group, increasing the donor strength of the OPE and promoting vectorial excitation energy transfer (EET) toward the porphyrin acceptor and it contributes to the amphiphilic balance of the molecule, facilitating controlled aggregation in aqueous media while maintaining π–π stacking within the hydrophobic porphyrin cores. This design was inspired by prior work on amino-OPE glycosides in photodynamic therapy and bioimaging, where the amino functionality supports hydrogen bonding and enhances photostability.^7–9^ The molecular design aimed to achieve vectorial EET , facilitating the directed flow of energy in a single preferred direction, a key challenge in molecular engineering.^10^ While natural photosystems exploit free-energy gradients and electronic coupling to induce efficient vectorial energy and electron transfer, synthetic analogue systems often struggle to achieve similar efficiency despite major advancements.^11,12^ The carbohydrate pendant moiety was incorporated to facilitate the formation of extended aggregates through hydrogen bonding interactions, and explore its potential applications in photodynamic therapy,^7,8^ fluorescent biomarking,^9^ or bacterial growth inhibition.^13^

Building on these previous findings, this study explores the excited-state dynamics of a gluco-Ammino-oligophenylenethylene-Porphyrin system (**GAP**) (Figure 1a) using transient absorption (TA) spectroscopy to characterize its monomeric (m**GAP**) and aggregated (a**GAP**) states. By comparing the ultrafast dynamics of m**GAP** and a**GAP**, we aim to elucidate how aggregation influences electronic relaxation pathways, energy transfer processes, and overall photostability. The observed differences in excited-state lifetimes and EET mechanisms align with previous studies on OPE-porphyrin assemblies, further emphasizing the pivotal role of supramolecular organization in tuning photophysical behavior.

**Figure 1:**
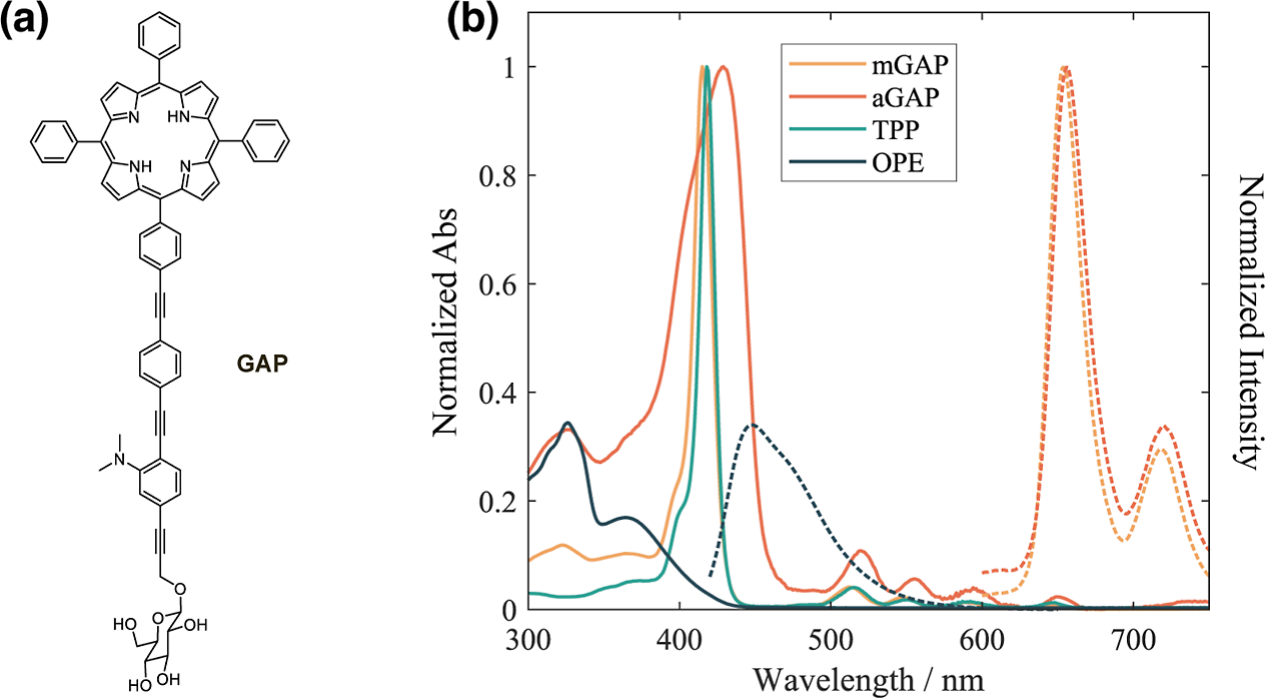
(a) Molecular structure of the monomeric form of **GAP**. (b) Normalized absorption (solid lines) and fluorescence emission (dashed lines) spectra of the monomeric (m**GAP**, yellow) in chloroform and aggregated (a**GAP**, orange) in methanol/water 60/40 v/v, compared with the absorption spectra of TPP (green) and OPE (blue) in chloroform.

## 2 Experimental and Theoretical Methods

### 2.1 Experimental Setup for TA Spectroscopy

The setup used for the TA measurements has been described previously.^14,15^ Briefly, a Ti/sapphire laser system (Mai-Tai and Spitfire, Spectra Physics) operating at 1 kHz repetition rate generated pulses centered at 800 nm with a 150 fs fwhm. The laser output was split by a 4% beam splitter into two parts: the weaker fraction was used to generate a supercontinuum white light in a thin sapphire plate, while the more intense fraction was focused onto a BBO crystal to produce a 400 nm pump pulse via second-harmonic generation. The pump repetition rate was reduced to 500 Hz using an optical chopper. The white-light probe (470–700 nm) was delayed with respect to the pump by means of a motorized linear translation stage that controlled the optical path length, and then split into two beams by a beam splitter. One portion of the white light served as a reference, while the other was directed onto the sample as the probe. The transmitted probe and reference beams were collected by a linear CMOS diode array detector. The signal-to-noise ratio was improved by repeating and averaging 100 (TPP and m**GAP**) and 200 (a**GAP**) measurements. The obtained spectra were processed to minimize white light chirping effects by using a homemade Matlab routine. Details on the procedures used for global fitting and kinetic analyses are provided in the Supporting Information.

### 2.2 Computational Details

Born–Oppenheimer molecular dynamics (BOMD) simulations were performed in the ground state using the ωB97X-D/6–31G­(d,p) level of theory as implemented in Gaussian 16. The simulations were carried out for a total duration of 1 ps with a time step of 0.5 fs at a constant temperature of 300 K.

To simplify the model, the d-glucoside group, known not to significantly influence the system’s photophysical properties, was replaced with an alcohol moiety.

Representative configurations were extracted from the BOMD trajectory, and for each of them, vertical excitation energies and oscillator strengths were calculated at the same level of theory to simulate the UV–Vis absorption spectrum. The spectrum at each wavelength was generated by assuming Gaussian lineshapes with a full width at half-maximum of 300 cm^–1^ for all transitions centered at the corresponding excitation wavelengths.

## 3 Results and Discussion

In good solvents such as methanol, the **GAP** molecule (Figure 1a) remains in solution as a monomer. However, in more polar methanol/water mixtures, the system undergoes self-assembly into large rod-like J-aggregates, likely driven by hydrogen bonding interactions between the sugar pendant arms.^16^ These aggregates exhibit aggregation-driven spectral shifts and modifications in electronic interactions, which are expected to significantly impact EET and relaxation pathways.

Figure 1b compares the absorption and fluorescence spectra of the individual components (TPP and OPE) with those of m**GAP** in methanol and a**GAP** in a 60/40 methanol/water mixture. In the visible range, the m**GAP** absorption spectrum (yellow) closely overlaps with that of the porphyrin moiety, featuring a B-band at 420 nm and four Q-bands between 500 and 700 nm. In agreement with previous literature,^17–19^ the four peaks in this spectral region can be attributed to transitions of the porphyrin moiety from the ground state to Q_x_(0) (645 nm), its vibrational sideband Q_x_(1) (590 nm), Q_y_(0) (548 nm), and Q_y_(1) (513 nm), respectively. The presence of the OPE unit is indicated by an additional low-intensity feature in the UV region. The fluorescence emission spectrum exhibits the characteristic two-band pattern of porphyrins, with maxima at 654 and 718 nm. Previous characterizations reported a fluorescence quantum yield of 0.33 and a monoexponential time-resolved fluorescence decay with a long lifetime of 8 ns, consistent with the typical behavior of porphyrins in their monomeric form.^6^

In the methanol/water mixture (orange), all bands undergo a red shift and broadening, as typically expected upon aggregate formation. This effect is particularly pronounced for the B-band, which has the highest oscillator strength. Aggregate formation in methanol/water mixtures was thoroughly investigated in previous studies. The presence of aggregates was confirmed by fluorescence quenching, AFM imaging, and resonance light scattering measurements, which indicated the involvement of at least 25 monomeric units in exciton formation.^6,20,21^

The absorption spectrum of the monomer was simulated using quantum mechanical methods to gain a fundamental understanding of the electronic transitions, enable accurate prediction of spectral features, and assist in the interpretation of experimental data by accounting for molecular structure, electron distribution, and environmental effects. Figure 2a shows the predicted absorption spectrum obtained for the m**GAP** model (orange stick spectrum) and compares it with the experimental spectrum. The computational results closely match the experimental spectrum, successfully reproducing the positions of the main absorption bands and validating the accuracy of the chosen theoretical approach. It should be noted that the TD-DFT calculations account only for purely electronic transitions and do not include vibronic coupling. Consequently, the vibronic transitions observed experimentally at 590 nm and 513 nm are not predicted in the simulated spectrum. Nonetheless, the model successfully reproduces the most important electronic features of the system.

**Figure 2:**
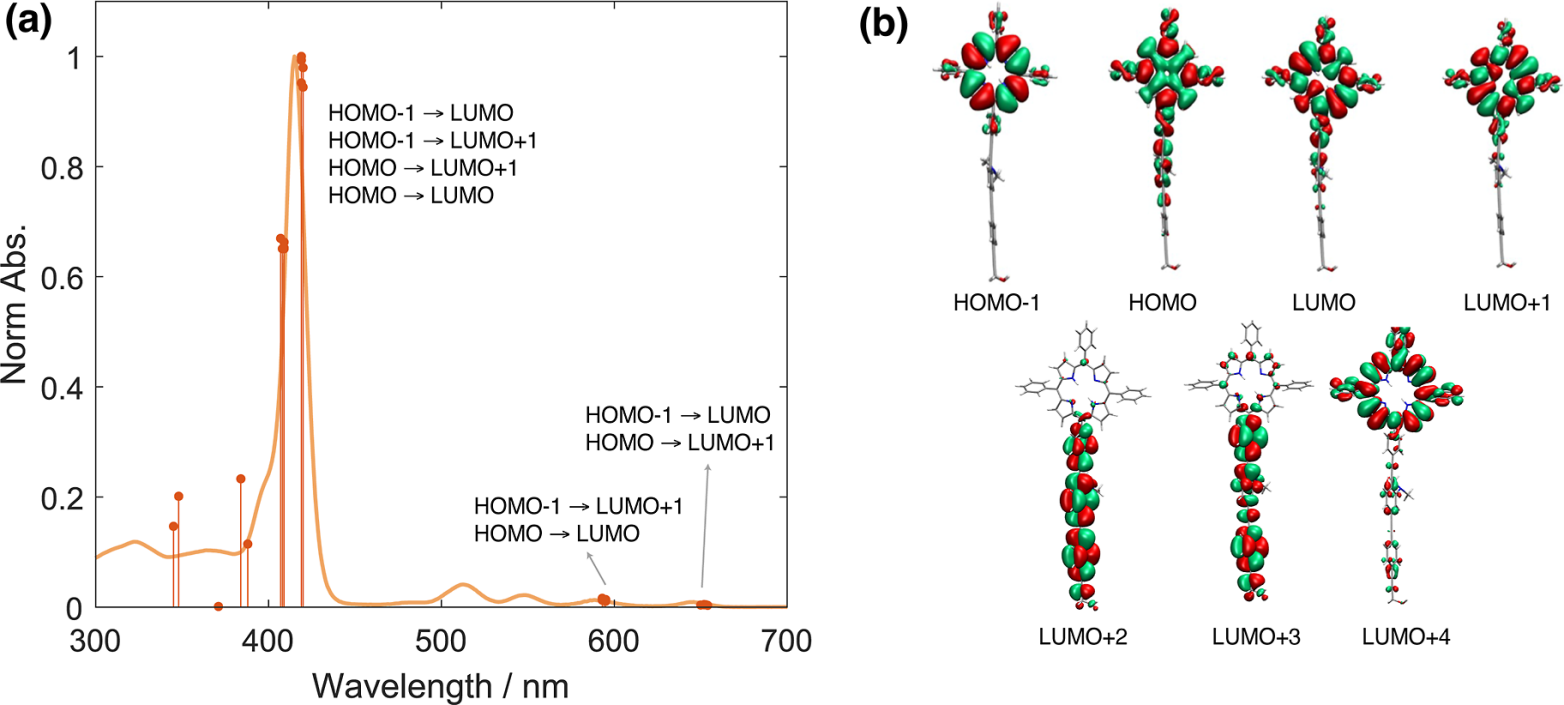
(a) Absorption spectrum computed at ωb97xd/6–31G­(d,p) level of theory for m**GAP** (orange bars) compared with the experimental spectrum (yellow line). (b) Frontier orbitals involved in the main energy electronic transitions. The green region represents the negative phase of the orbitals, while the red region represents the positive phase.

Further analysis identifies the key frontier orbitals contributing to the main electronic transitions. It was found that HOMO–1, HOMO, LUMO, and LUMO+1 primarily contribute to the transitions responsible for the B- and Q-bands in the absorption spectrum. In contrast, higher-energy transitions involve orbitals with clear localization on either the OPE or TPP moiety. These orbitals are illustrated in Figure 2b.

The excited state dynamics of the two species were investigated using TA spectroscopy. The TA signal is expressed as the differential absorption Δ*A*(*t*,λ) as a function of the probe wavelength λ and the time delay *t* after pump excitation. This can be represented as two-dimensional plots, as shown in Figure 3. Δ*A*(*t*,λ)∝ – (*I*(*t*,λ) – *I*_0_(λ))/*I*_0_(λ), where *I*_0_(λ) and *I*(*t*,λ) are the intensity of the signal at probe wavelength λ without pump excitation and at a time delay *t* after pump excitation, respectively. Negative Δ*A* signals are the result of stimulated emission (SE) and ground-state bleaching (GSB), while positive Δ*A* signals are the result of excited state absorption (ESA) to higher lying excited states.

**Figure 3:**
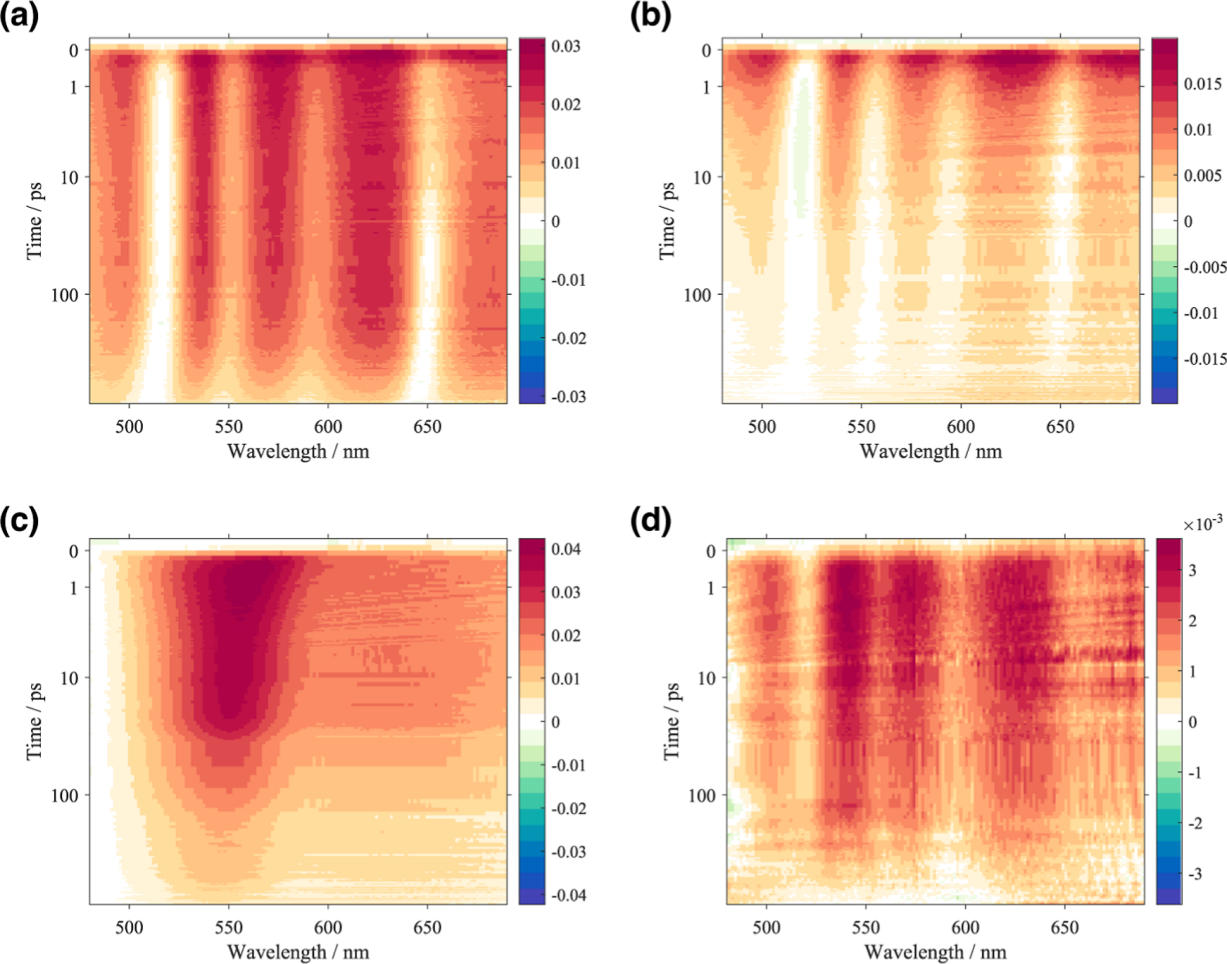
Two-dimensional transient absorption spectra plotting the differential absorbance Δ*A*(*t*,λ) as a function of the probe wavelength (λ) and the time delay (*t*, in logarithmic scale). The responses of the two samples are presented: (a) m**GAP** in methanol and (b) a**GAP** in methanol/water 60/40, and compared with control samples: (c) OPE in chloroform and (d) TPP in methanol/chloroform 50/50.

Figure 3 compares the two-dimensional TA spectra of the studied samples, m**GAP** and a**GAP**, with those of the control samples: the unfunctionalized TPP molecule in 50/50 chloroform/methanol and OPE in chloroform, measured under the same experimental conditions.

The results obtained from the detailed analysis of the m**GAP** sample are summarized in Figure 4. Figure 4a compares the absorption spectrum of m**GAP** in the 480–690 nm range with its TA spectra, measured at selected time delays after excitation. These TA spectra correspond to horizontal slices of the two-dimensional response shown in Figure 3a at the chosen time delays. In this spectral region, the TA spectrum is characterized by a broad positive background generated by ESA signals from the Q states. This positive band shows a series of local minima due to the overlap of negative GSB contributions of the Q bands, approximately at the same wavelengths where the Q transitions appear in the absorption spectrum. The local minimum at 650 nm is deeper and slightly red-shifted compared to expectations from the absorption spectrum due to the contribution of the SE at 654 nm, which adds to the GSB contribution at 645 nm.

**Figure 4:**
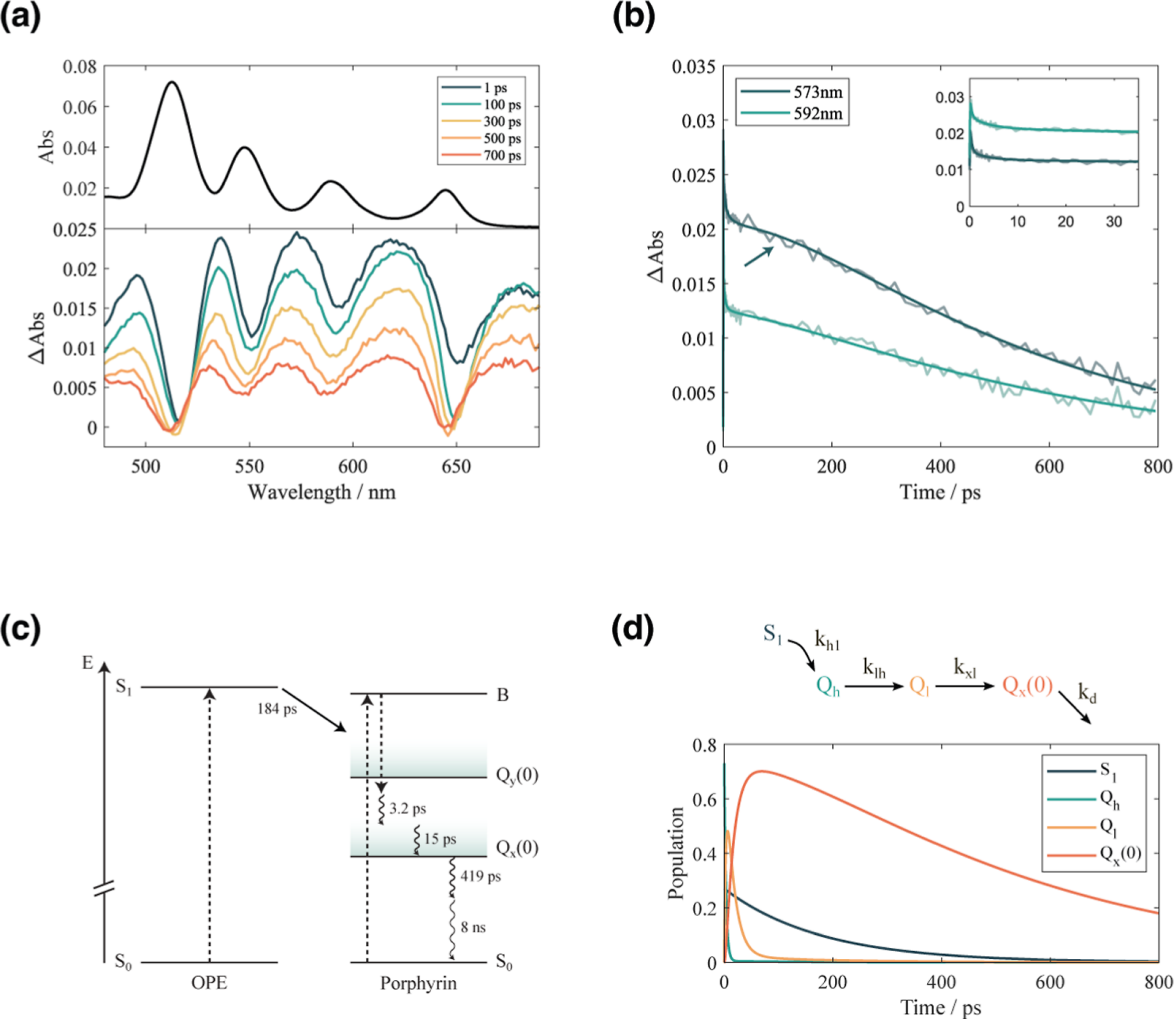
(a) Absorption spectrum (top) and TA spectra at selected time delays after excitation (bottom) of m**GAP** in methanol, in the 480–690 nm spectral region. (b) Temporal evolution of the TA signal of m**GAP** at two relevant probe wavelengths (573 and 592 nm). The arrow indicates the rise of the TA signal occurring after the first hundred ps, characterized by a time constant of 184 ps. The inset shows the first 35 ps with greater detail. (c) Schematic representation of the photophysics of the system that results from the interpretation of the fittings. Dashed arrows represent phenomena occurring on a time scale comparable to the time resolution of the experiment; the solid arrow represents the energy transfer; wavy arrows represent relaxation processes. (d) The kinetic model used to interpret the time evolution of the TA response (top) and the corresponding time evolution of the kinetic species (bottom).

The temporal dynamics can be visualized better by plotting Δ*A* at a specific λ as a function of *t*. This corresponds to taking vertical slices of the two-dimensional response shown in Figure 3a at the selected wavelengths. As an example, Figure 4b presents the temporal traces extracted at 573 and 592 nm; however, similar trends are observed at other relevant wavelengths.

The temporal traces at all wavelengths were fitted using a global multiexponential model with a shared set of time constants, enabling a consistent description of the different decay components across the studied spectral range and providing insight into the underlying relaxation processes^22,23^ (see also Section S1 for further details on the fitting procedures). For m**GAP**, the early time dynamics (<100 ps) closely resemble those of the unfunctionalized TPP molecule (see Supporting Information, Sections S2 and S3) and are characterized by rapidly decaying components with time constants of 0.3, 3.2, and 15 ps.

The 0.3 ps time constant can be attributed to a combination of ultrafast processes occurring on a time scale comparable to the experimental time resolution. Since internal conversion from the Soret and Q_y_ bands of the porphyrin to the Q_x_ band is expected to occur within this time frame^17,24^ the 0.3 ps component likely encompasses all relaxation processes by which the excitation created on the porphyrin moiety transitions to the lowest-energy Q_x_ band.

The 3.2 and 15 ps time constants are instead consistent with vibrational relaxation within the Q_x_ band, as similar time scales have been reported for free-base and substituted tetraphenylporphyrin molecules.^17,25,26^ Overall, these processes efficiently funnel population into the lowest vibrational level of the Q_x_ state within tens of picoseconds following photoexcitation in the Soret band.

Over longer times (>100 ps), in addition to a time component exceeding the experimental time window —likely due to the ns emission lifetime of the lowest Q_x_ state, in agreement with previous time-resolved fluorescence experiments^6,27^ —the temporal dynamics of m**GAP** are governed by two additional components with time constants of 184 and 419 ps (see also Table S2).

The 419 ps decay component may be attributed to a relaxation of the Q_x_(0) state preceding the ns emission. Indeed, a relaxation trend with a time constant of the same order of magnitude (272 ps) is also seen in the time evolution of the TA signal of isolated TPP (see Figure S1 and Table S1), used as a reference. This dynamic is too slow to be attributed to solvent relaxation, which is expected to occur within tens of ps.^28,29^ Instead, the time scale of this relaxation could be compatible with conformational changes in the excited-state geometry, already observed in diacid porphyrins^27^ or rotational diffusion.^31^

The 184 ps time constant is the most intriguing feature, as it was not observed in the time evolution of TPP. This time constant is associated with a rising kinetic component (i.e., it is characterized by a negative amplitude in the global fitting parameters;^22,23^ see Table S2). It accounts for the increase in the TA signal amplitude observed in the decay traces of m**GAP** after the first hundred picoseconds, as indicated by the arrow in Figure 4b. A rise in the TA signal over time indicates the presence of a dynamic process that populates the states corresponding to the wavelengths at which the time trace was extracted,^32^ which in this case are the Q-band states.

Unlike the TPP case, where the 400 nm excitation promotes high-energy states belonging to the B band manifold, in m**GAP**, the excitation at 400 nm is expected to also populate the S_1_ state of the OPE moiety. Thus, it is reasonable to initially hypothesize that the rise time detected in m**GAP** may be linked to an EET process transferring population from the OPE to the porphyrin moiety. The overall interpretation that results from the multiexponential analysis is schematically summarized in Figure 4c.

This assignment is supported by TD-DFT simulations, which show that the high-energy transitions i.e. LUMO+1, LUMO+2, LUMO+3 involve orbitals localized on the OPE moiety, excited by the 400 nm pump (see also Supporting Information, Section S5).

To quantify the contribution of EET from the OPE to the time-dependent population of Q_x_(0), a kinetic model was developed (Figure 4d). In this model, we define *k*_h1_ as the rate constant of EET from the S_1_ state of the OPE to a high-energy state within the Q bands of the porphyrin (denoted as Q_h_ for generality). Since excitation within the Q bands of the porphyrin relaxes into the Q_x_ band within the time resolution of the experiment, it is not possible to determine whether Q_h_ belongs to the Q_y_ band or represents a vibrationally excited state of Q_x_.

From Q_h_, the system undergoes vibrational relaxation to lower-energy states within the Q_x_ manifold (denoted as Q_l_) before eventually reaching Q_x_(0) with rate constants *k*_lh_ and *k*_xl_, respectively. Finally, the signal of Q_x_(0) decays with *k*_d_.

As shown in the bottom panel of Figure 4d, the temporal evolution of the kinetic species associated with Q_x_(0) matches the long-term trend of the signal recorded at 573 nm. The initial rise in Q_x_(0), which results from the rapid relaxation of excitation directly deposited on the porphyrin by the pump pulse, is not explicitly visible in the time traces at 536, 573, and 621 nm. This is due to its overlap with the rapidly decaying excited-state absorptions from higher-energy Q bands (not included in the model).

The model provides a semiquantitative estimate indicating that approximately 70% of the population reaching the Q_x_(0) state arises from direct excitation of the porphyrin moiety, while the remaining ∼30% is transferred from the OPE. Despite the limitations in the precision of this estimate, the analysis suggests that a non-negligible portion of the excitation energy funnelled into the lowest porphyrin states is initially harvested by the OPE. In constructing the kinetic model in Figure 4d, we started by assuming that the initial acceptor state of energy from the OPE was a high-energy Q-band state. Since, based on spectral overlap considerations, the presence of alternative acceptor states, such as the B bands or lower energy states of the B bands, cannot be excluded, the model was extended to also explore alternative initial acceptor states. This further analysis (reported in Section S4 of the Supporting Information) confirms that temporal evolution of the population on the final Q_x_(0) state is not significantly influenced by the state chosen as initial acceptor, as fast relaxation processes from B to Q bands and within the Q-band manifold ensure that energy ultimately efficiently reaches Q_x_(0), regardless of the pathway taken (see Figure S2).

Figure 5 summarizes the dynamics of aggregated **GAP** (a**GAP**) obtained in a 60/40 methanol/water mixture. TA spectra for a**GAP** were recorded under the same experimental conditions as m**GAP**. The TA spectrum of a**GAP** exhibits the same peak pattern as m**GAP**, with only a slight red-shift of the Q-band GSBs observed, consistent with the red-shift of the Q transitions noted in the absorption spectrum of a**GAP**. However, substantial differences can be detected in the temporal dynamics of the signals, as shown in Figure 5b. The application of a multiexponential model, in addition to a long-time decaying component reflecting the sample’s emission in the ns regime,^6^ revealed three distinct decay components of 1, 10, and 116 ps (Supporting Information, Table S3).

**Figure 5:**
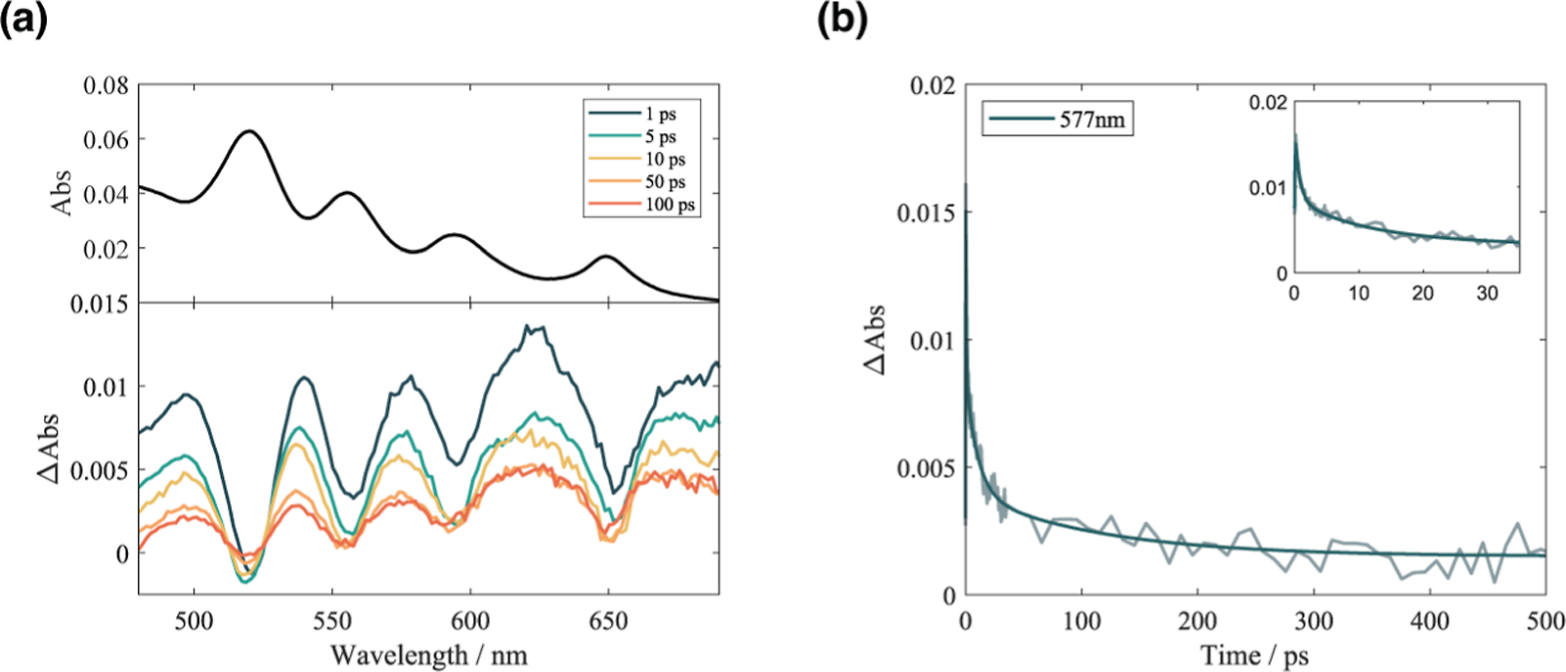
(a) TA spectra at selected time delays after excitation of a**GAP** in a 60/40 methanol/water mixture. (b) Temporal evolution of the TA signal of a**GAP** at a relevant probe wavelength (577 nm). The inset shows the first 35 ps with greater detail.

The spectral features in the TA spectra are essentially identical for m**GAP** and a**GAP**, indicating that the hydrophobic porphyrin cores are sequestered within the aggregate interior, while the hydrophilic glycosidic groups are likely exposed to the aqueous environment, in agreement with previous investigations.^6^ This structural arrangement preserves the fundamental electronic character of the porphyrin units while enabling new intermolecular interactions that significantly alter their relaxation dynamics. Indeed, the similarity in spectral profiles suggests that the time constants governing the dynamics of a**GAP** correspond to the same relaxation processes identified for m**GAP**, but proceed on markedly faster time scales.

An acceleration of the overall dynamics upon aggregation is a well-known feature of porphyrins and has been reported for several functionalized tetraphenylporphyrin systems.^30,33–36^ However, our findings not only confirm this acceleration but also provide a quantitative elucidation of how aggregation reshapes the excited-state dynamics. This detailed mechanistic picture goes beyond qualitative descriptions of “quenching” and reveals how specific supramolecular interactions modulate electronic relaxation pathways—an issue central to physical chemistry. In particular, the faster excited-state decay observed here can be attributed to the formation of extended excitonic networks through π–π stacking and hydrogen-bonding interactions, which introduce new nonradiative relaxation channels and promote exciton delocalization across multiple chromophoric units.^30,37^ This supramolecular organization effectively modifies the electronic structure, lowering the energy barriers for internal conversion and enabling rapid dissipation of excitation energy.^38^ Such cooperative effects account for the observed fluorescence quenching and shortened excited-state lifetimes.^6^

No rise components were detected in the dynamics of the aggregated sample, making it impossible to directly trace the EET from OPE to TPP moieties, as observed in the monomeric species. This may imply that the excitation of the OPE follows a different pathway in the aggregated sample and/or that detecting the spectral signature of the EET would require a shorter time resolution due to the overall shortening of the time scales in a**GAP**.

A particularly interesting aspect of this context is that, in supramolecular aggregated structures stabilized by hydrogen bonding, H-bonds are not merely structural elements but also play a crucial role in shaping the electronic structure from both static and dynamic perspectives.^39,40^ On one hand, hydrogen bonding interactions can modulate the static electronic properties of molecules, such as transition dipole moments, transition energies, and electronic couplings.^14^ On the other hand, these interactions also influence dynamic electronic properties by altering charge distribution, significantly affecting ultrafast excited-state dynamics.^41^ This modulation has profound implications for the efficiency and mechanisms of various photophysical processes, including photoinduced electron transfer reactions in biological photosynthesis,^42,43^ artificial photosynthesis,^44^ and organic photovoltaics.^2^ Such dynamics typically unfold on ultrafast time scales, often within hundreds of femtoseconds.

While the TA measurements reported here lack the temporal resolution needed to directly resolve these processes, they strongly suggest that if these processes occur, they must happen within this ultrafast regime. Such behavior parallels that of natural light-harvesting complexes, where strong intermolecular coupling and hydrogen bonding drive ultrafast, directional energy flow. This further supports the potential of a**GAP** as a promising material for achieving efficient and directional EET.

## 4 Conclusions

In conclusion, the ultrafast response of a porphyrin-based system monosubstituted with a spectroscopically active group reveals time-dependent spectroscopic features that are absent in the porphyrin moiety alone. This indicates significant interactions between the two components, with analysis suggesting possible EET from the OPE unit to the lower-energy states of the porphyrin. Such a system effectively mimics the energy funneling capabilities of natural photosynthetic complexes by absorbing energy across different regions of the visible spectrum and directing it toward lower-energy electronic states.

These findings provide a foundational step toward the design of advanced systems in which the sugar moiety can be replaced by functional groups tailored for applications in energy conversion or chemiluminescent materials. Our results show that the presence of the sugar does not significantly affect the photophysical behavior of the system, while remaining essential for the formation of biocompatible aggregates.

In the aggregated state, the **GAP** system exhibits markedly accelerated relaxation dynamics, reflecting the emergence of new nonradiative pathways induced by supramolecular organization. These intermolecular interactions not only stabilize the aggregate structure but also modulate its electronic landscape. The ultrafast relaxation observed in the aggregated forms poses challenges for characterization, as key processes likely occur on time scales beyond the temporal resolution of the present measurements. Future studies employing higher time resolution will be essential to resolve these initial relaxation steps and fully elucidate the energy transfer mechanisms at play. Nonetheless, the present findings indicate that **GAP** aggregates emulate the functional principles of natural light-harvesting complexes, where cooperative excitonic coupling and hydrogen-bond-mediated interactions enable rapid and directional energy flow. These insights underscore the potential of porphyrin–OPE aggregates as promising building blocks for the rational design of supramolecular materials with tunable excited-state dynamics for optoelectronic and photonic applications.

## Supplementary Material



## Notes

Additional experimental details, fitting procedure, kinetic analysis, computational details, and additional Transient Absorption figures (PDF)
