# Supplementary material for: Ultrafast Excited-State Dynamics of a Porphyrin–OPE Conjugate: Energy Transfer and Aggregation Effects
Source: J Phys Chem C Nanomater Interfaces. 2025 Nov 26;129(49):21720–7. doi: 10.1021/acs.jpcc.5c07515 (PMC12707222; doi:10.1021/acs.jpcc.5c07515)
Supplement: Supplementary file 1 [file jp5c07515_si_001.pdf]

Supporting Information

Ultrafast Excited-State Dynamics of a Porphyrin–OPE Conjugate: Energy Transfer and Aggregation Effects

Francesco Tumbarello,[1] Federico Toffoletti,[1] Chiara Maria Antonietta Gangemi,[2] Mariagrazia Fortino,[3] Adriana Pietropaolo,[3] Anna Barattucci,[2] Luigi Monsù Scolaro,[2] Maria Angela Castriciano,[2] Elisabetta Collini[1,]*

[1] Department of Chemical Sciences, University of Padova, via Marzolo 1, 35131 Padova, Italy

[2] Dipartimento di Scienze Chimiche, Biologiche, Farmaceutiche ed Ambientali (ChiBioFarAm), Università degli Studi di Messina, 98166 Messina, Italy

[3] Dipartimento di Scienze della Salute, Università di Catanzaro, Viale Europa, 88100 Catanzaro, Italy

*elisabetta.collini@unipd.it

S1. Fitting procedure

The analysis of the temporal traces extracted at different wavelengths was performed by using as fitting function $f(t)$ a sum of exponential functions convoluted with a Gaussian function having FWHM = 0.15 ps and centered at t = 0:

$$f(t) = \left(\frac{1}{w\sqrt{2\pi}} e^{-\frac{t^2}{2w^2}}\right) * \left(\sum_i A_i e^{-\frac{t}{\tau_i}}\right) = \sum_i \frac{A_i}{2} e^{-\frac{1}{\tau_i}\left(t-\frac{w^2}{2\tau_i}\right)} \left[1 + erf\left(\frac{t-\frac{w^2}{\tau_i}}{w\sqrt{2}}\right)\right] \qquad \text{Eq S1}$$

where w is linked to the FWHM of the Gaussian as $w = \frac{FWHM}{2\sqrt{2ln2}}$.

The temporal traces were fitted globally: while the same set of time constants was shared between all the traces, the amplitudes were allowed to be optimized independently. Global fitting analysis is often used in pump-probe spectroscopy to extract fundamental kinetic and spectral parameters by analyzing the entire data set (across multiple wavelengths) simultaneously, rather than fitting individual time traces. It was proven to be a particularly suitable method for complex systems because it yields a single set of rate constants that describes all observed dynamics consistently, providing a deeper understanding of the underlying photochemical or photophysical processes.[1,2]

Singular value decomposition analysis was applied to determine the minimal set of parameters necessary to achieve a robust and accurate reproduction of the experimental dynamics.[2]

The results of the fittings performed using the model of Eq. S1 at selected wavelengths are summarized in Section S2 for the unfunctionalized TPP molecule, and in Section S3 for mGAP and aGAP, respectively.

S2. Analysis of unfunctionalized TPP

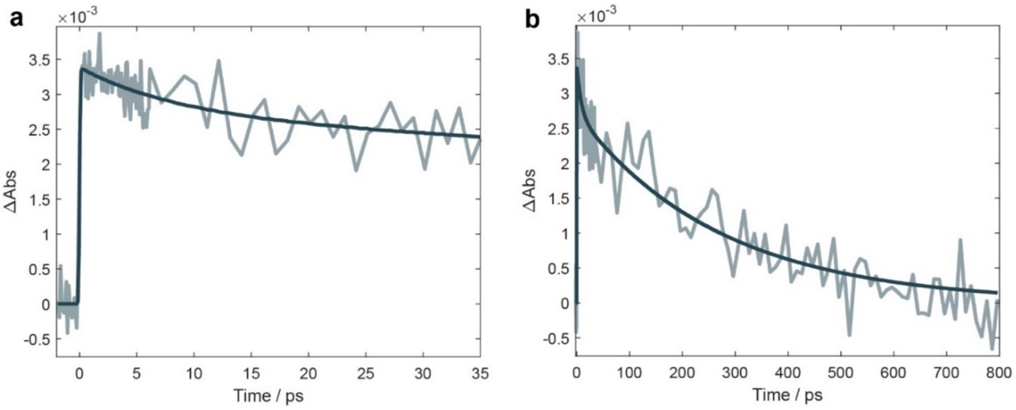

Figure S1. *Fitting of the temporal trace of TPP in a methanol/chloroform 50/50 mixture, extracted at 539 nm.*

**λ / nm**	τ_1 = **8.5** ps **A₁**	τ_2 = **272.2** ps **A₂**
**539**	0.20	0.80
**573**	0.17	0.83
**630**	0.20	0.80

Table S1. *Time constants and relative amplitudes retrieved by the global fitting of the temporal traces of TPP extracted at selected wavelengths.*

S3. Results of the global fitting for mGAP and aGAP

**λ / nm**	τ_1 = **0.3** ps A_1	τ_2 = **3.2** ps A_2	τ_3 = **14.6** ps A_3	τ_4 = **184.1** ps A_4	τ_5 = **418.6** ps A_5
**536**	0.09	0.07	0	-0.21	0.63
**551**	0.09	0.03	0.01	-0.01	0.52
**573**	0.09	0.04	0.03	-0.26	0.58
**592**	0.14	0.04	0.01	-0.07	0.51
**621**	0.15	0	0.02	-0.28	0.55

Table S2. *Time constants and relative amplitudes retrieved by the global fitting of the temporal traces of mGAP at selected wavelengths. Positive amplitudes represent the decay of positive signals, while negative amplitudes represent a rise of positive signals or the decay of negative signals.*

λ / nm	τ_1 = **0.8** ps<br>**A_1**	τ_2 = **10.3** ps<br>**A_2**	τ_3 = **115.7** ps<br>**A_3**	τ_4 > **800** ps<br>**A_4**
**538**	0.37	0.32	0.24	0.07
**577**	0.49	0.28	0.15	0.09
**623**	0.55	0.25	0.08	0.12

Table S3. *Time constants and relative amplitudes retrieved by the global fitting of the temporal traces of aGAP at selected wavelengths.*

The error on the experimental values has been estimated to be about 10% from repeated measurements.

S4. Kinetic Analysis

As an aid to correct interpretation of the TA data, different kinetic models were designed and compared.

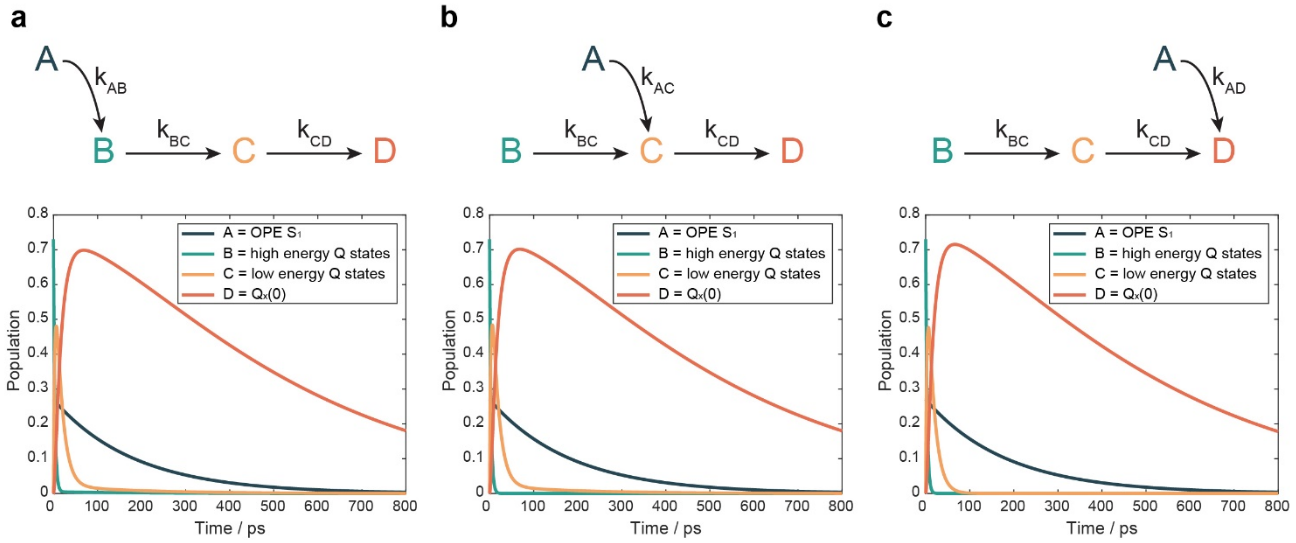

Figure S2. *Comparison between kinetic models differing for the energy transfer pathway.*

The species indicated with letters A to D shown in Figure S3.1 represent, respectively: (A) the S_1 state of OPE, (B, C) vibrationally excited states in the manifold of Q_x and (D) $Q_x(0)$. In the proposed scheme, only A and B have nonnull population at time t = 0 (A_0 and B_0): indeed, OPE is directly excited by the pump pulse at 400 nm, and the population deposited by the pump in the Soret band of the porphyrin relaxes into the Q_x manifold within the time resolution of the experiment. k_{AB} is the rate constant of the energy transfer from the OPE to the Q bands, k_{BC} and k_{CD} are the rate constants of vibrational relaxation processes occurring within the Q_x manifold and k_{DE} is the relaxation rate

constant of $Q_x(0)$. It is convenient, for the purpose of discussion, to define the combination of rate constants $k_1, k_2, \ldots, k_6$ as follows:

$$k_1 = \frac{k_{AB}}{k_{AB}-k_{BC}},\ k_2 = \frac{k_{BC}}{k_{CD}-k_{BC}},\ k_3 = \frac{k_{BC}}{k_{CD}-k_{AB}},\ k_4 = \frac{k_{CD}}{k_{DE}-k_{BC}},\ k_5 = \frac{k_{CD}}{k_{DE}-k_{CD}},\ k_6 = \frac{k_{CD}}{k_{DE}-k_{AB}}$$

The expressions of the temporal evolution of the kinetic species can therefore be written as

$$A(t) = A_1 e^{-k_{AB}t}$$

$$\text{With } A_1 = A_0$$

$$B(t) = B_1 e^{-k_{BC}t} + B_2 e^{-k_{AB}t}$$

$$\text{With } B_1 = B_0 + A_0 k_1,\ B_2 = -A_0 k_1$$

$$C(t) = C_1 e^{-k_{BC}t} + C_2 e^{-k_{CD}t} + C_3 e^{-k_{AB}t}$$

$$\text{With } C_1 = B_0 k_2 + A_0 k_1 k_2,\ C_2 = -B_0 k_2 - A_0 k_1 k_2 + A_0 k_1 k_3,\ C_3 = -A_0 k_1 k_3$$

$$D(t) = D_1 e^{-k_{BC}t} + D_2 e^{-k_{DE}t} + D_3 e^{-k_{CD}t} + D_4 e^{-k_{AB}t}$$

$$\text{With } D_1 = B_0 k_2 k_4 + A_0 k_1 k_2 k_4,\ D_2 = -B_0 k_2 k_4 + B_0 k_2 k_5 - A_0 k_1 k_2 k_4 + A_0 k_1 k_2 k_5 + A_0 k_1 k_3 k_6 - A_0 k_1 k_3 k_5,\ D_3 = -B_0 k_2 k_5 - A_0 k_1 k_2 k_5 + A_0 k_1 k_3 k_5,\ D_4 = -A_0 k_1 k_3 k_6$$

These equations were used to calculate the curves represented in Figure S3.1, using the time constants obtained through the fitting discussed in the main text. Since the first and the third members in the expression of $D(t)$ are relevant only at early times (< 100 ps), at longer times $D(t)$ can be approximated as:

$$D(t) \approx D_2 e^{-k_{DE}t} + D_4 e^{-k_{AB}t}$$

The amplitude of the ESA signal from the $Q_x(0)$ state must be proportional to the population in this state. Indicating with k the proportionality constants:

$$ESA_{Q_x(0)} \approx kD_2 e^{-k_{DE}t} + kD_4 e^{-k_{AB}t} = A_5 e^{-t/\tau_5} + A_4 e^{-t/\tau_4}$$

$A_5 = kD_2$ and $A_4 = kD_4$ are the amplitudes of the long decay and late rise components of the multiexponential model used to fit the traces at 536, 573 and 621 nm, as discussed in the main text. The ratio between the amplitudes A_4/A_5, obtained by the fitting, must thus correspond to the ratio between the amplitudes D_4/D_5 predicted by the kinetic model. The D_4/D_5 ratio equals the A_4/A_5 ratio for $A_0 = 0.27$ and $B_0 = 0.73$. This provides an approximate estimation of the relative population that arrives into the $Q_x(0)$ state after the energy transfer from the OPE.

By considering the overlap between emission from the OPE and absorption of Q states of the porphyrin moiety, it is reasonable to hypothesize that the excitation is funneled from the S_1 state of the OPE to the Q_y band. The energy then relaxes into the Q_x band within the time resolution of the experiment. It can be shown, however, that even if the transfer occurred to a lower energy state of the porphyrin, the description of the system would remain almost unchanged. The comparison between the kinetic models schematically represented in Figure S3.1 demonstrates that the temporal behavior of the species corresponding to $Q_x(0)$ is not significantly affected by the choice of the state to which the OPE transfers the excitation, since relaxations into $Q_x(0)$ are much faster than the energy transfer.

S5. Additional Computational Details

The starting coordinates of the monomeric GAP system were generated by replacing the D-glucoside moiety with an alcohol group. Those coordinates were optimized at the ωB97xd/6-31G(d,p) level of theory in the ground state using the *Gaussian16* software.

Analysis of the orbital contributions indicates that the HOMO is localized both on porphyrin and OPE, whereas HOMO–1 is largely localized on the porphyrin macrocycle, with negligible OPE contribution. In contrast, orbitals localized on the OPE moiety appear instead at deeper occupied levels. The high-energy transitions populating LUMO+1, LUMO+2, and LUMO+3 (with significant OPE character, particularly for the latter two) therefore mainly originate from OPE-localized occupied orbitals. This result supports the experimental interpretation that excitations addressed by the 400 nm pump are best described as having predominant OPE character. This assignment is fully consistent with the experimental transient absorption data, which reveal that excitation initially deposited on the OPE subsequently undergoes energy transfer to the porphyrin Q states.

S6. Supplementary References

(1) van Stokkum, I. H. M.; Larsen, D. S.; van Grondelle, R. Global and Target Analysis of Time-Resolved Spectra. *Biochim. Biophys. Acta BBA - Bioenerg.* **2004**, *1657* (2), 82–104. https://doi.org/10.1016/j.bbabio.2004.04.011.

(2) Volpato, A.; Bolzonello, L.; Meneghin, E.; Collini, E. Global Analysis of Coherence and Population Dynamics in 2D Electronic Spectroscopy. *Opt. Express* **2016**, *24* (21), 24773–24785. https://doi.org/10.1364/oe.24.024773.
